# Development and validation of A CT-based radiomics nomogram for prediction of synchronous distant metastasis in clear cell renal cell carcinoma

**DOI:** 10.3389/fonc.2022.1016583

**Published:** 2023-01-04

**Authors:** Xinxin Yu, Lin Gao, Shuai Zhang, Cong Sun, Juntao Zhang, Bing Kang, Ximing Wang

**Affiliations:** ^1^ School of Medicine, Shandong University, Jinan, China; ^2^ Department of Radiology, Shandong Provincial Hospital Affiliated to Shandong First Medical University, Jinan, China; ^3^ Department of Nuclear Medicine, The First Affiliated Hospital of Shandong First Medical University & Shandong Provincial Qianfoshan Hospital, Jinan, China; ^4^ School of Medicine, Shandong First Medical University, Jinan, China; ^5^ GE Healthcare, PDx GMS Advanced Analytics, Shanghai, China

**Keywords:** radiomics, nomogram, clear cell renal cell carcinoma, metastasis, computed tomography

## Abstract

**Background:**

Early identification of synchronous distant metastasis (SDM) in patients with clear cell Renal cell carcinoma (ccRCC) can certify the reasonable diagnostic examinations.

**Methods:**

This retrospective study recruited 463 ccRCC patients who were divided into two cohorts (training and internal validation) at a 7:3 ratio. Besides, 115 patients from other hospital were assigned external validation cohort. A radiomics signature was developed based on features by means of the least absolute shrinkage and selection operator method. Demographics, laboratory variables and CT findings were combined to develop clinical factors model. Integrating radiomics signature and clinical factors model, a radiomics nomogram was developed.

**Results:**

Ten features were used to build radiomics signature, which yielded an area under the curve (AUC) 0.882 in the external validation cohort. By incorporating the clinical independent predictors, the clinical model was developed with AUC of 0.920 in the external validation cohort. Radiomics nomogram (external validation, 0.925) had better performance than clinical factors model or radiomics signature. Decision curve analysis demonstrated the superiority of the radiomics nomogram in terms of clinical usefulness.

**Conclusions:**

The CT-based nomogram could help in predicting SDM status in patients with ccRCC, which might provide assistance for clinicians in making diagnostic examinations.

## Introduction

Renal cell carcinoma (RCC) represents the seventh most prevalent malignant tumors, leading to around 140,000 deaths every year (1). Clear cell RCC (ccRCC) is the major histological subtype, accounting for about 80% of all cases (2). Owing to the widespread use of advantage radiologic diagnostic techniques, as well as the popularization in regular checkups, most incidentally detected renal lesions are small low-grade tumors. Nevertheless, 20%-30% of ccRCC patients have distant metastases at the time of diagnosis (synchronous distant metastasis, SDM) (3). Surgery is no longer suitable for metastatic ccRCC due to widespread metastatic disease; thus, systemic therapy is applicable in this setting (4, 5). ccRCC with SDM has poor prognosis, with median survival of 16 months and a five-year survival rate of 3.6% (3). Early identification of SDM can certify the reasonable, personalized, and efficient treatment strategies were timely performed and ultimately improve patient survival (6). Hence, it was of great value to estimate the possibility of combined distant metastasis, by which we can fully make individualized examination and treatment plans.

Several clinicopathological parameters have been identified to establish the nomogram for predicting SDM of ccRCC patients (7, 8): T stage, pathological differentiation grade, lymph node status, tumor size, and the invasion beyond the capsule. One of the most meaningful risk factors is the tumor size of the primary tumor. However, even small ccRCC have the potential to present SDM (9, 10), which is mean that relying too heavily on tumor size can lead to underestimate the true incidence of SDM. Many advanced imaging manners can contribute to the detection of SDM. However, using multitudinous imaging methods to check all potential metastatic sites for every ccRCC patient will heighten the extra economic and physical burden. On the other hand, some metastatic lesions may be small or share the overlapping imaging characteristics with other tumors, which can lead to the risk of missed diagnosis or misdiagnosis even though imaging examinations were performed (11–14).

Radiomics is a promising technique using computerized quantitative imaging analysis to extract an enormous quantity of image-related features, such as intensity, geometry, and texture, from medical images (15, 16). Radiomics features extracted from computed tomography (CT), and magnetic resonance imaging (MRI) have been successfully applied in predict SDM in ccRCC patients (17, 18). However, these models were developed with limited samples or without external validation, making their clinical usefulness very limited. Moreover, clinical risk factors, which could improve predictive accuracy, have been overlooked.

In this multicenter study, we aim to develop and validate a CT-based radiomics nomogram, incorporating radiomics signature and clinical risk factors, for preoperative prediction of SDM in patients with ccRCC, based on a large collection of patient data from two different institutions.

## Materials and methods

### Patients

**Figure 1:**
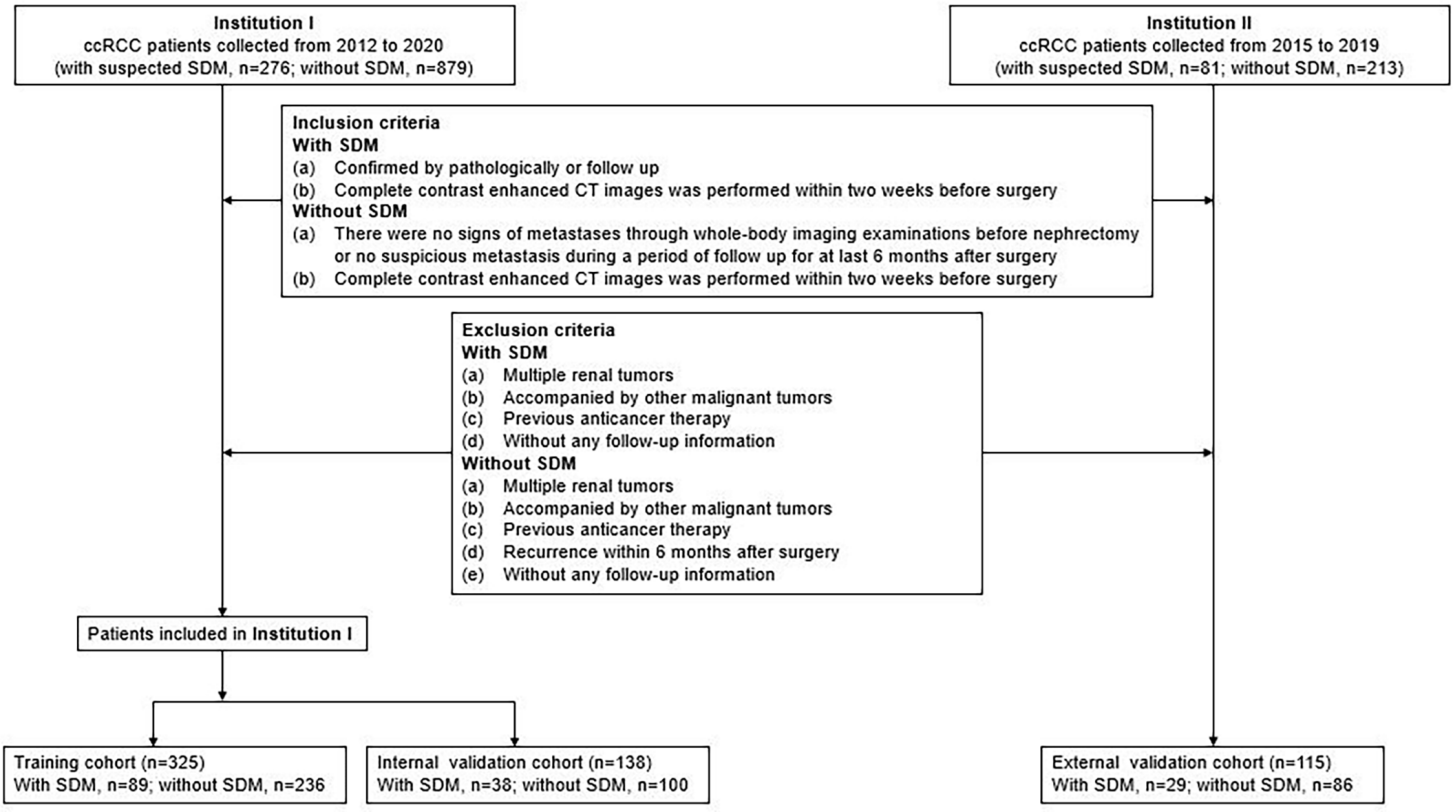
Recruitment pathway for patients in this study. ccRCC, clear cell renal cell carcinoma.

The study was conducted in accordance with the Declaration of Helsinki (as revised in 2013). This retrospective study was approved by the Institutional Review Board of Shandong Provincial Hospital Affiliated to Shandong First Medical University and individual consent for this retrospective analysis was waived. The study population flowchart is illustrated in **Figure 1**.

Data for surgically and pathologically confirmed ccRCC cases were acquired by searching through the institutional database and medical record system. The inclusion and exclusion criteria of the patients are presented in **Supplementary S1**. Four hundred sixty-three patients from Shandong Provincial Hospital Affiliated to Shandong First Medical University diagnosed between January 2012 to December 2020, including 127 SDM ccRCC patients and 336 without SDM ccRCC patients, were randomly assigned to either the training cohort and internal validation cohort in a 7:3 ratio, using a stratified random split in patient level. External validation cohort consisted of 115 patients from Shandong Medical Imaging Research Institute between January 2015 to December 2019, including 29 SDM ccRCC patients and 86 without SDM ccRCC patients. A total of 58 SDM were confirmed by pathology, and the other SDM were diagnosed by radiologic features, that is, there was an increase in volume or number of suspected metastases during follow-up. SDM was defined as the distant metastatic lesion existing at the time of initial diagnosis before nephrectomy.

Demographic and clinical characteristics, including age, gender, weight, coronary heart disease, diabetes, hypertension, history of smoking, hemoglobin, red blood cell distribution width (RDW), neutrophil count, lymphocyte count, platelet count, creatinine, calcium, albumin, fibrinogen were derived from medical records. Besides, we also calculated neutrophil-to-lymphocyte ratio (NLR), platelet-to-lymphocyte ratio (PLR), RDW-to-lymphocyte ratio (RLR), and albumin-to-fibrinogen ratio (AFR).

### CT image acquisition and radiologic evaluation

The details of image acquisition parameters are shown in **Supplementary S2**. Each CT study was analyzed by a radiology resident (Reader 1, BK) and a radiologist (Reader 2, XMW) with 5 and 20 years of experience in abdominal imaging, respectively. Aware of the diagnosis of ccRCC but blinded to the radiological reports and pathologic details, the two researchers construed the following CT features by consensus: the maximum diameter of tumor on the axial CT image; tumor polarity (superior/middle/inferior); tumor side (left/right); tumor margin (well defined/poorly defined); tumor shape (round/lobulated/irregular); enhancement degree (lower than cortex/higher than or similar to cortex); and necrosis (absence/presence). The maximum diameter of the tumor was measured by the two radiologists, and the average value was applied to the evaluation. For those qualitative parameters, in the event of disagreement, the two readers jointly reviewed the findings to reach a consensus for further analysis.

### Development of clinical factor model

Univariate regression analysis was applied to the clinical factors, including clinical data (age, gender, weight, coronary heart disease, diabetes, hypertension, history of smoking), laboratory variables (hemoglobin, PLR, NLR, RLR, AFR, calcium, and creatinine), and CT features to find the factor that significantly affected the event occurrence probability. Then a multiple logistic regression analysis with a step-wise backwards elimination was subsequently applied to build the clinical factors model in the training cohort. Odds ratios (OR) as estimates of relative risk with 95% confidence interval (CI) were calculated for each risk factor.

### Segmentation of tumor images and radiomics feature extraction

In order to remove the potential differences of CT images acquired from different CT scanners, normalization was performed on all original CT images using the gray-scale discretization method before extracting the radiomics features.

Corticomedullary phase and nephrographic phase images at 5.0-mm thickness were retrieved for radiomics feature extraction. The three-dimensional region of interest (ROI) were manually segmented along the tumor contour on each transverse section, avoiding covering the paratumoral renal parenchyma and perinephric fat, by using RadCloud (Huiying platform Medical Technology Co., Ltd.), which was an available platform reliably used in previous studies. Finally, 1409 radiomics feature were extracted, detailed in **Supplementary S3**.

Inter- and intra-class correlation coefficients (ICCs) were calculated to estimate the inter-observer reliability and intra-observer reproducibility of features extraction. Fifty cases of CT images containing 17 SDM ccRCCs and 33 without SDM ccRCCs were randomly chosen; region-of-interest segmentation was drawn by one radiology resident (Reader 1, BK) and one radiologist (Reader 2, XMW) independently; both were aware of the diagnosis of ccRCC but were blinded to the SDM status. Reader 1 then repeated the contouring procedure 8 weeks after the initial analysis to assess the agreement of feature extraction. The remaining image segmentation was performed by Reader 1.

### Development of radiomics signature and radiomics nomogram

Only were the radiomics chosen to be kept when meeting a criterion of inter- and intra-observer ICCs greater than 0.75, then the minimum redundancy maximum relevancy method was performed to eliminate the redundant and irrelated features and kept 30 features. The remaining features were enrolled into the least absolute shrinkage and selection operator (LASSO) regression model to choose the optimized subset of features from the training cohort to construct the final model. A radiomics model was created by summing the selected feature values weighted by their respective coefficients, and the corresponding radiomics score was calculated for each patient.

To provide a more individualized predictive model, a nomogram combining the final radiomics model and clinical factors model was built in the training cohort. The calibration of the nomogram was evaluated with a calibration curve. The Hosmer–Lemeshow test was conducted to assess the goodness-of-fit of the nomogram. A radiomics nomogram score for each patient was obtained in the testing and external validation cohorts.

### Assessment of the performance of different models

The predictive accuracy of the clinical factors model, radiomics model, and radiomics nomogram for predicting SDM were quantified by the area under the receiver operating characteristics (ROC) curve (AUC). Decision curve analysis (DCA) was used to calculate the net benefits for a range of threshold probabilities in the whole cohort to assess the clinical usefulness of the nomogram.

### Statistical analysis

Statistical analysis were performed using R statistical software (version 3.6.3, https://www.r-project.org). Group differences of the clinical factors were figured out by means of chi-square test or Fisher exact test for categorical variables and Mann-Whitney U test for continuous variables, where appropriate. The clinical factors model was constructed using the backward step-wise multivariate logistic regression with Akaike information criterion (AIC) as criterion. The LASSO logistic regression was performed using the “glmnet” package; the ROC curves were plotted using the “pROC” package; the nomogram and calibration curves were performed using the “rms” package; and the DCA was performed using “rmda” package.

## Results

### Clinical factors of the patients and construction of the clinical factor model

The patients’ demographic baseline characteristics are summarized in **Table 1**. There are 325 ccRCC patients in the training cohort (216 men and 109 women; mean age, 55.3 ± 11.1 years), 138 patients in the internal validation cohort (95 men and 43 women; mean age, 55.4 ± 10.4 years) and 115 patients in the external validation cohort (84 men and 31 women; mean age, 53.9 ± 10.9 years). The rates of SDM ccRCCs were 27.4% (89 of 325), 27.5% (38 of 138), and 25.2% (29 of 115) in the training, internal validation, and external validation cohorts, respectively, whereas no statistically significant difference was found among the three cohorts (*P*=0.891). The confirmation approaches of SDM and sites of metastases are shown in **Table 2**.

**Table 1:** Comparison of patient clinicoradiological characteristics of with and without SDM ccRCC.

Characteristics	Training cohort			Internal validation cohort			External validation cohort		
	With SDM (n=89)	Without SDM (n=236)	*P* Value	With SDM (n=38)	Without SDM (n=100)	*P* Value	With SDM (n=29)	Without SDM (n=86)	*P* Value
Age (y)^*^	58.5 ± 9.2	54.1 ± 11.5	0.001	57.9 ± 10.0	54.5 ± 10.4	0.082	56.8 ± 10.2	52.9 ± 11.0	0.093
Sex			<0.001			0.889			0.524
Female	12 (13.5)	97 (41.1)		11 (28.9)	32 (32.0)		6 (20.7)	25 (29.1)	
Male	77 (86.5)	139 (58.9)		27 (71.1)	68 (68.0)		23 (79.3)	61 (70.9)	
Hypertension			0.524			0.826			0.555
Absence	54 (60.7)	154 (65.3)		22 (57.9)	54 (54.0)		19 (65.5)	49 (57.0)	
Presence	35 (39.3)	82 (34.7)		16 (42.1)	46 (46.0)		10 (34.5)	37 (43.0)	
Diabetes			0.573			1.000			0.185
Absence	79 (88.8)	202 (85.6)		33 (86.8)	88 (88.0)		27 (93.1)	69 (80.2)	
Presence	10 (11.2)	34 (14.4)		5 (13.2)	12 (12.0)		2 (6.9)	17 (19.8)	
Coronary heart disease			0.442			0.852			0.684
Absence	77 (86.5)	213 (90.3)		36 (94.7)	92 (92.0)		26 (89.7)	81 (94.2)	
Presence	12 (13.5)	23 (9.7)		2 (5.3)	8 (8.0)		3 (10.3)	5 (5.8)	
History of smoking			0.248			0.279			1.000
Absence	52 (58.4)	156 (66.1)		21 (55.3)	67 (67.0)		58 (67.4)	19 (65.5)	
Presence	37 (41.6)	80 (33.9)		17 (44.7)	33 (33.0)		28 (32.6)	10 (34.5)	
Weight^*^	70.8 ± 11.3	71.5 ± 12.2	0.654	70.4 ± 11.5	74.2 ± 10.5	0.067	72.3 ± 12.3	68.5 ± 12.9	
Hemoglobin (g/L)			<0.001			<0.001			0.001
Female≥115; Male≥130	54 (60.7)	218 (92.4)		24 (63.2)	95 (95.0)		21 (72.4)	83 (96.5)	
Female<115; Male<130	35 (39.3)	18 (7.6)		14 (36.8)	5 (5.0)		8 (27.6)	3 (3.5)	
Calcium (mmol/L)			0.043			0.875			0.056
≤2.4	53 (59.6)	170 (72.0)		25 (65.8)	69 (69.0)		12 (41.4)	55 (64.0)	
>2.4	36 (40.4)	66 (28.0)		13 (34.2)	31 (31.0)		17 (58.6)	31 (36.0)	
Creatinine (μmol/L)			0.014			0.299			0.866
≤90	76 (85.4)	223 (94.5)		32 (84.2)	92 (92.0)		25 (86.2)	71 (82.6)	
>90	13 (14.6)	13 (5.5)		6 (15.8)	8 (8.0)		4 (13.8)	15 (17.4)	
RLR			<0.001			0.022			0.648
<8.6	45 (50.6)	179 (75.8)		22 (57.9)	79 (79.0)		20 (69.0)	65 (75.6)	
≥8.6	44 (49.4)	57 (24.2)		16 (42.1)	79 (79.0)		9 (31.0)	21 (24.4)	
PLR			<0.001			<0.001			0.008
<146	29 (32.6)	140 (59.3)		12 (31.6)	72 (72.0)		10 (34.5)	56 (65.1)	
≥146	60 (67.4)	96 (40.7)		26 (68.4)	28 (28.0)		19 (65.5)	30 (34.9)	
NLR			<0.001			<0.001			0.004
<3	51 (57.3)	199 (84.3)		18 (47.4)	85 (85.0)		18 (62.1)	76 (88.4)	
≥3	38 (42.7)	37 (15.7)		20 (52.6)	15 (15.0)		11 (37.9)	10 (11.6)	
AFR			<0.001			<0.001			<0.001
≥9	42 (47.2)	218 (92.4)		14 (36.8)	98 (98.0)		10 (34.5)	82 (95.3)	
<9	47 (52.8)	18 (7.6)		24 (63.2)	2 (2.0)		19 (65.5)	4 (4.7)	
Maximum diameter (mm)^*^	76.2 ± 27.2	46.8 ± 18.5	<0.001	70.5 ± 26.0	42.0 ± 13.7	<0.001	73.6 ± 29.8	42.3 ± 14.2	<0.001
Tumor side			0.517			1.000			0.479
Left	45 (50.6)	108 (45.8)		18 (47.4)	46 (46.0)		14 (48.3)	50 (58.1)	
Right	44 (49.4)	128 (54.2)		20 (52.6)	54 (54.0)		15 (51.7)	36 (41.9)	
Tumor polarity			0.693			0.304			0.035
Superior	30 (33.7)	71 (30.1)		12 (31.6)	29 (29.0)		13 (44.8)	20 (23.3)	
Middle	31 (34.8)	94 (39.8)		12 (31.6)	45 (45.0)		7 (24.1)	42 (48.8)	
Inferior	28 (31.5)	71 (30.1)		14 (36.8)	26 (26.0)		9 (31.0)	24 (27.9)	
Tumor margin			<0.001			<0.001			<0.001
Well defined	34 (38.2)	213 (90.3)		17 (44.7)	95 (95.0)		11 (37.9)	82 (95.3)	
Poorly defined	55 (61.8)	23 (9.7)		21 (55.3)	5 (5.0)		18 (62.1)	4 (4.7)	
Tumor shape			<0.001			<0.001			<0.001
Round	11 (12.4)	103 (43.6)		4 (10.5)	47 (47.0)		3 (10.3)	25 (29.1)	
Lobulated	18 (20.2)	95 (40.3)		15 (39.5)	43 (43.0)		10 (34.5)	51 (59.3)	
Irregular	60 (67.4)	38 (16.1)		19 (50.0)	10 (10.0)		16 (55.2)	10 (11.6)	
Enhancement degree			0.476			0.453			0.910
High	71 (79.8)	198 (83.9)		30 (78.9)	86 (86.0)		23 (79.3)	71 (82.6)	
Low	18 (20.2)	38 (16.1)		8 (21.1)	14 (14.0)		6 (20.7)	15 (17.4)	
Necrosis			<0.001			<0.001			0.006
Absence	22 (24.7)	148 (62.7)		11 (28.9)	70 (70.0)		15 (51.7)	69 (80.2)	
Presence	67 (75.3)	88 (37.3)		27 (71.1)	30 (30.0)		14 (48.3)	17 (19.8)	
Rad-score^†^	0.1 [-0.9, 1.5]	-2.2 [-2.8, -1.2]	<0.001	-0.6 [-1.4, 1.0]	-2.4 [-3.0, -1.5]	<0.001	0.4 [-0.5, 1.8]	-1.8 [-2.3, -1.1]	<0.001

Unless otherwise indicated, data are numbers of patients, and data in parentheses are percentages. ^*^Data are mean ± standard deviation. ^†^Data in parentheses are interquartile range.

**Table 2:** Confirmation approaches of SDM and sites of metastases.

	Total (n=156)	Training cohort (n=89)	Internal validation cohort (n=38)	External validation cohort (n=29)
Number of metastasis sites				
Single	105 (67.3%)	64 (71.9%)	20 (52.6%)	21 (72.4%)
Multiple	51 (32.7%)	25 (28.1%)	18 (47.4%)	8 (27.6%)
Sites of metastases				
Lung	93 (59.6%)	52 (58.4%)	26 (68.4%)	15 (51.7%)
Bone	53 (34.0%)	27 (30.3%)	18 (47.4%)	8 (27.6%)
Adrenal gland	27 (17.3%)	14 (15.7%)	7 (18.4%)	6 (20.7%)
Lymph nodes	27 (17.3%)	17 (19.1%)	7 (18.4%)	3 (10.3%)
Liver	13 (8.3%)	8 (9.0%)	4 (10.5%)	1 (3.4%)
Pleura	10 (6.4%)	4 (4.5%)	4 (10.5%)	2 (6.9%)
Brain	9 (5.8%)	4 (4.5%)	3 (7.9%)	2 (6.9%)
Pancreas	5 (3.2%)	4 (4.5%)	–	1 (3.4%)
Others^*^	5 (3.2%)	2 (2.2%)	–	3 (10.3%)
Confirmation approaches				
By pathology	58 (37.2%)	31 (34.8%)	13 (34.2%)	14 (48.3%)
By follow-up	98 (62.8%)	58 (65.2%)	25 (65.8%)	15 (51.7%)

Data are numbers of patients, and data in parentheses are percentages. ^*^Other sites included the bladder, gall bladder, muscles and spleen.

The results of multiple logistic regression analysis are listed in **Table 3**. According to the backward step-wise multivariate logistic regression, age, sex, maximum diameter, shape, margin, calcium, hemoglobin, and AFR were incorporated into the development of the clinical factor model. The clinical score (Cli-score) was calculated with the following formula:

**Table 3:** Multivariate logistic regression analysis of clinicoradiological characteristics.

	OR (95% CI)	*P* Value
Age	1.04 (1.00,1.08)	0.076
Male	4.99 (1.9,13.15)	0.001
Decreased hemoglobin	2.3 (0.77,6.88)	0.137
Elevated calcium	2.88 (1.24,6.69)	0.014
Elevated creatinine	1.11 (0.24,5.22)	0.892
Elevated RLR	0.72 (0.27,1.95)	0.521
Elevated PLR	1.23 (0.47,3.22)	0.680
Elevated NLR	0.84 (0.3,2.36)	0.740
Decreased AFR	14.94 (5.44,41.08)	<0.001
Maximum diameter	1.04 (1.01,1.06)	0.003
Poorly defined	2.32 (0.86,6.25)	0.096
Lobulated	0.39 (0.13,1.23)	0.109
Irregular	2.06 (0.61,6.97)	0.244
Necrosis	1.67 (0.68,4.09)	0.264


$$\begin{array}{l} Cliscore=0.029 * age + 1.359 * sex + maximum * 0.036 + shape * 0.456\\ +\text{ margin }*\;1.102\;+\;calcium * 0.957 + hemoglobin * 0.944 + AFR\\ {}* 2.442-8.159 \end{array}$$

**Figure 2:**
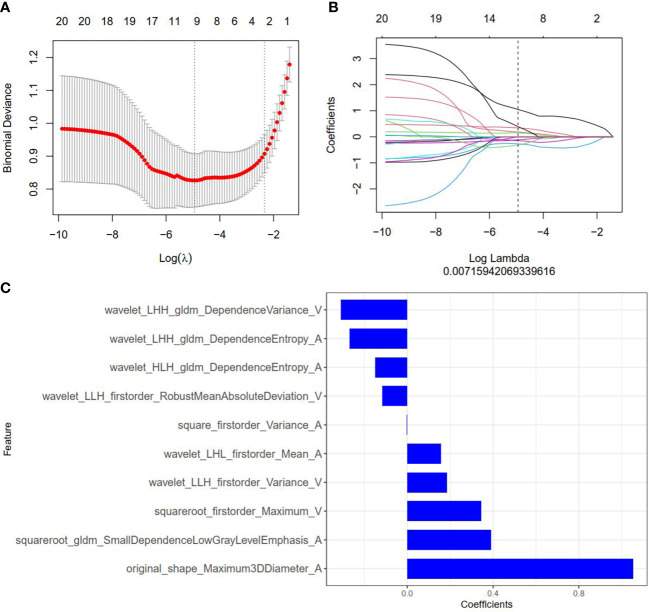
Radiomics feature selection by using the least absolute shrinkage and selection operator (LASSO) logistic regression. **(A)** Selection of the tuning parameter (λ) in the LASSO model via 10-fold cross-validation based on minimum criteria. Binomial deviances from the LASSO regression cross-validation model are plotted as a function of log(λ). The y-axis shows binomial deviances and the lower x-axis the log(λ). Numbers along the upper x-axis indicate the average number of predictors. Red dots indicate average deviance values for each model with a given λ, and vertical bars through the red dots indicate the upper and lower values of the deviances. The vertical black lines define the optimal values of λ, where the model provides its best fit to the data. An optimal λ value of 0.007 with log(λ) =-4.962 was selected. **(B)** The coefficients have been plotted vs. log(λ). **(C)** The 10 features with nonzero coefficients are shown in the plot.

ROC curves of clinical factors model are displayed in **Figure 2**, which yielded an AUC of 0.924 (95% CI: 0.890, 0.959) in the training cohort, 0.896 (95% CI: 0.826, 0.966) in the internal validation cohort, and 0.920 (95% CI: 0.850, 0.990) in the external validation cohort.

### Radiomics feature extraction, selection, and radiomics signature establishment

**Figure 3:**
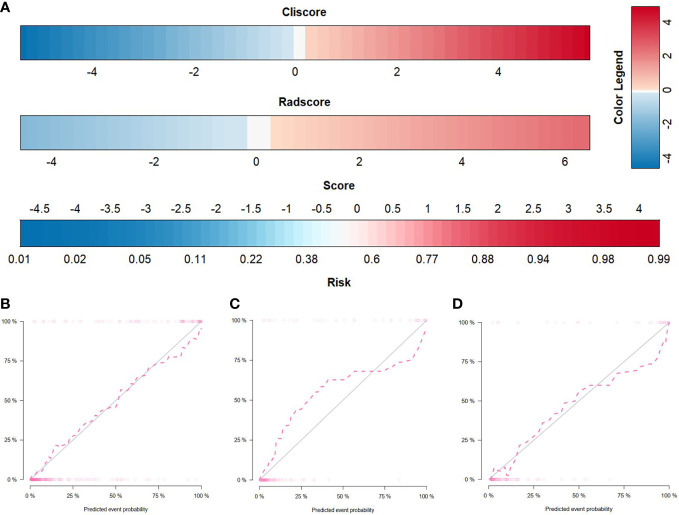
Radiomics nomogram and calibration curves. **(A)** The radiomics nomogram, combining Cli-score and Rad-score, developed in the training set. Calibration curves for the radiomics nomogram in the training **(B)**, internal validation **(C)**, and external validation **(D)** cohorts. Calibration curves indicate the goodness-of-fit of the nomogram. The 45° gray line represents the ideal prediction, and the pink line represents the predictive performance of the nomogram. The closer the pink line approaches the ideal prediction line, the better the predictive efficacy of the nomogram is.


Among 2818 radiomics features extracted from corticomedullary phase and nephrographic phase CT images, 1704 features showed high stability, and then were reduced to 30 features by minimum redundancy maximum relevancy. In the final feature selection with the LASSO method (**Figures 3A, B**), 10 most valuable features were kept, and displayed in **Figure 3C**. Violin plots showed that the difference of the 10 radiomics features between the SDM ccRCC and without SDM ccRCC groups (**Supplementary Figure S1**). The radiomics score (Rad-score) was attained with the following formula:

$$\begin{array}{l} Radscore=1.051 * original\_shape\_Maximum3DDiameter\_A+ -0.268\\ {}* wavelet\_LHH-gldm\_DependenceEntropy\_A + 0.183\\ {}* wavelet\_LLH\_firstorder\_Variance\_V + 0.156\\ {}* wavelet\_LHL\_firstorder\_Mean\_A + -0.148\\ {}* wavelet\_HLH\_gldm\_DependenceEntropy\_A + 0.343\\ {}* squareroot\_firstorder\_Maximum\_V + -0.309\\ {}*wavelet\_LHH\_gldm\_DependenceVariance\_V + -0.002\\ {}* square\_firstorder\_Variance\_A + -0.115\\ {}* wavelet\_LLH\_firstorder\_RobustMeanAbsoluteDeviation\_V + 0.391\\ {}*squareroot\_gldm\_SmallDependenceLowGrayLevelEmphasis\_A + -1.335 \end{array}$$

Rad-score [median (interquartile range)] differed significantly between the SDM ccRCC and without SDM ccRCC groups in the training cohort [0.1 (-0.9, 1.5) *vs*. −2.2 (−2.8, −1.2), respectively, *P* < 0.001]; this finding was verified in the internal validation cohort [−0.6 (−1.4, 1.0) *vs*. −2.4 (−3.0, −1.5), respectively, *P* < 0.001] and external validation cohort [0.4 (−0.5, 1.8) *vs*. −1.8 (−2.3, −1.1), respectively, *P* < 0.001].

ROC curves of radiomics signature are displayed in **Figure 2**. The radiomics signature yielded an AUC of 0.871 (95% CI: 0.828, 0.914) in the training cohort, 0.869 (95% CI: 0.806, 0.933) in the internal validation cohort, and 0.882 (95% CI: 0.796, 0.967) in the external validation cohort, showing favorable predictive efficacy.

### The radiomics nomogram establishment and assessment of the performance of different models

**Figure 4:**
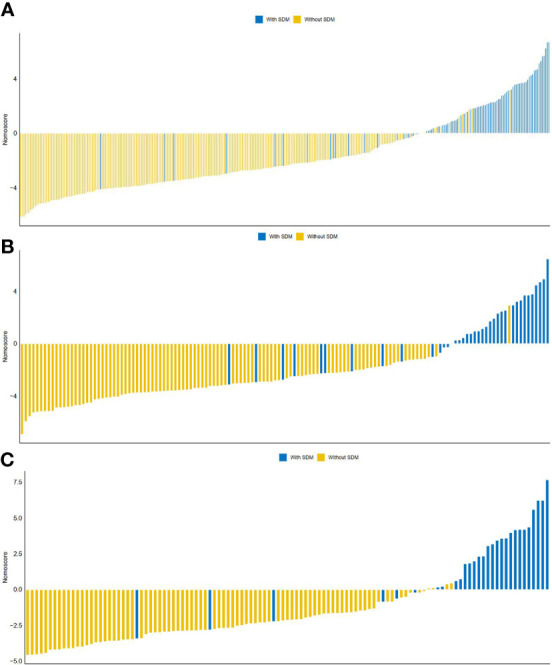
The distribution of Nomo-score with regard to SDM status in the training **(A)**, internal validation **(B)** and external validation **(C)** cohorts.

By incorporating the Cli-score and Rad-score, a radiomics nomogram was developed in the training cohort (**Figure 4A**). The calibration curve of the radiomics nomogram demonstrated good agreement between the predicted and expected probabilities for SDM ccRCC (**Figure 4**). The *P* values of Hosmer–Lemeshow test were 0.471, 0.183, and 0.340 in training, internal validation, and external validation cohorts, respectively.

**Figure 5:**
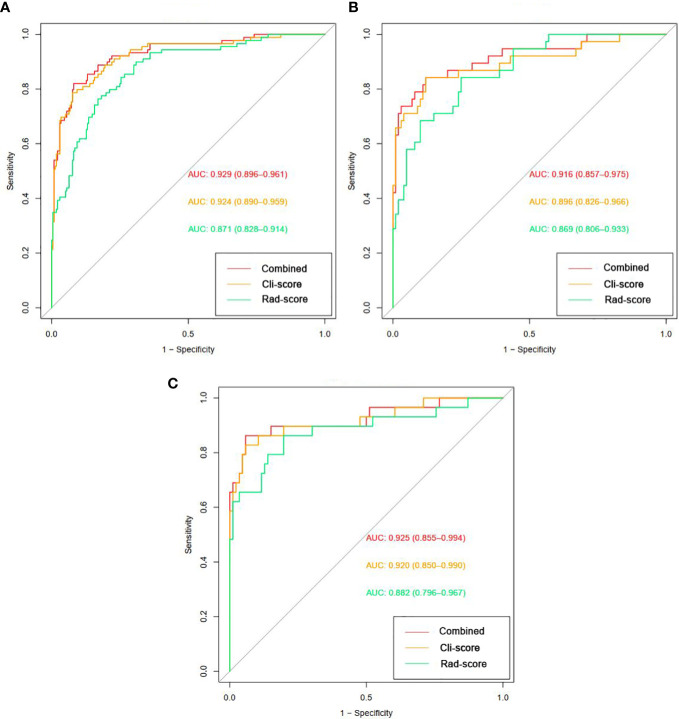
Diagnostic performance of the clinical factors model, radiomics signature, and radiomics nomogram was assessed and compared through ROC curves in the training **(A)**, internal validation **(B)** and external validation **(C)** cohorts. ROC = receiver operating characteristics; AUC = area under the receiver operating characteristic curve.

ROC curves of radiomics nomogram are displayed in **Figure 2**. The AUC, sensitivity, specificity, and accuracy of the radiomics nomogram, respectively, were 0.929 (95% CI: 0.896, 0.961), 82.0%, 91.9%, and 89.2% in the training cohort, 0.916 (95%CI: 0.857, 0.975), 84.2%, 88.0%, and 87.0% in the internal validation cohort, 0.925 (95%CI: 0.855,0.994), 86.2%, 94.2%, and 92.2% in the external validation cohort. The distribution of Nomo-score with regard to SDM status in the training, internal validation and external validation cohorts is presented in **Figure 5**.

The diagnostic performance of every model is demonstrated in **Table 4**. A slightly higher AUC was observed for the radiomics nomogram after integrating Cli-score both in the internal validation cohort (0.916 *vs*. 0.869) and in the external validation cohort (0.925 *vs*. 0.882). Nevertheless, incorporation of the Cli-score into the radiomics nomogram did not show significantly improved prediction efficiency (*P* =0.181 and 0.133, respectively).

**Table 4:** Results of radiomics nomogram, radiomics signature, and the clinical factors model predictive ability for distinguishing between SDM ccRCC and without SDM ccRCC.

Parameter		Cutoff	AUC (95% CI)	ACC*	SEN*	SPE*
Clinical factors model	Training cohort	-0.396	0.924 (0.890-0.959)	88.6% (288/325)	78.7% (70/89)	92.4% (218/236)
	Internal validation cohort		0.896 (0.826-0.966)	87.0% (120/138)	84.2% (32/38)	88.0% (88/100)
	External validation cohort		0.920 (0.850-0.990)	91.3% (105/115)	82.8% (24/29)	94.2% (81/86)
Radiomics signature	Training cohort	-0.884	0.871 (0.828-0.914)	81.2% (264/325)	76.4% (68/89)	83.1% (196/236)
	Internal validation cohort		0.869 (0.806-0.933)	77.5% (107/138)	84.2% (32/38)	75.0% (75/100)
	External validation cohort		0.882 (0.796-0.967)	81.7% (94/115)	86.2% (25/29)	80.2% (69/86)
Radiomics nomogram	Training cohort	0.419	0.929 (0.896-0.961)	89.2% (290/325)	82.0% (73/89)	91.9% (217/236)
	Internal validation cohort		0.916 (0.857-0.975)	87.0% (120/138)	84.2% (32/38)	88.0% (88/100)
	External validation cohort		0.925 (0.855-0.994)	92.2% (106/115)	86.2% (25/29)	94.2% (81/86)

Note.—CI, confidence interval.

$ACC=\frac{TP+\;TN}{TP\;+\;TN\;+\;FP\;+\;FN}$ $SEN=\frac{TP}{TP\;+\;FN}$ $ACC=\frac{TP\;+\;TN}{TP\;+\;TN\;+\;FP\;+\;FN}$ where TP, FP, TN, and FN denote true positive, false positive, true negative, and false negative, respectively.

*Numbers in parentheses were used to calculate percentages.

**Figure 6:**
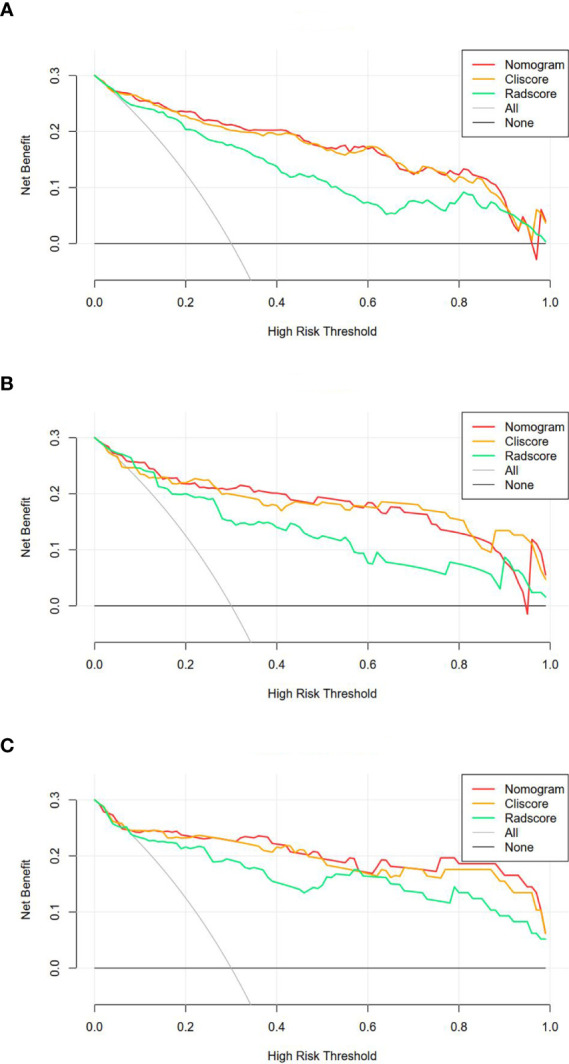
Decision curve analysis for the three models in the training **(A)**, internal validation **(B)** and external validation **(C)** cohorts. The y-axis shows the net benefit; x-axis shows the threshold probability. The red, orange, and green line represent net benefit of the radiomics nomogram, clinical factors model, and radiomics signature, respectively.

The DCA of the three model were presented in **Figure 6**. It showed that the radiomics nomogram and clinical factor model had a higher overall net benefit in differentiating SDM ccRCC from without SDM ccRCC than the radiomics signature across the full range of reasonable threshold probabilities.

## Discussion

It is necessary to preoperatively identify the SDM status timely to certify the reasonable, personalized, and efficient treatment decision. In this retrospective study, we developed and validated a radiomics nomogram that incorporates the radiomics signature and clinical factors for individualized prediction of SDM in ccRCC patients before treatment. The proposed radiomics nomogram demonstrated favorable discrimination in both internal validation cohort (AUC, 0.916) and external validation cohort (AUC, 0.925), outperforming radiomics signature (internal validation, 0.869; external validation, 0.882) and clinical factor model (internal validation, 0.896; external validation, 0.920).

As far as we know, only few studies have been reported in the literature including radiomics-based methods for prediction of SDM ccRCC. Bai et al. (17) developed a MRI-based radiomics nomogram combining patient age, regional lymph node, pseudocapsule and Rad-score, and demonstrated the nomogram can be useful for differentiating SDM ccRCC from without SDM ccRCC, with an AUC of 0.854 (95%CI, 0.736-0.971) in the internal validation cohort and 0.816 (95%CI, 0.661-0.971) in the external validation cohort. Compared with MRI, CT has a wider range of uses for the detection, identification, and staging of ccRCC due to its high diagnostic accuracy. A study by Wen et al. (18) showed that radiomics features extracted from contrast enhanced CT images demonstrated a good performance for prediction of SDM ccRCC (AUC, 0.83 [95%CI, 0.69-0.95]). However, the weakness of their study is lack of relevant clinical factors. Therefore, we tried to develop a novel CT-based radiomics nomogram combined radiomics signature and clinical factors to predict the SDM status. In addition, our study had a larger sample size with 578 ccRCC patients while the previous studies only had modest sample size ranging from 172 to 201 patients. Our study showed a better performance compared with previous studies, with an AUC of 0.916 (95%CI, 0.857-0.975) in the internal validation cohort and 0.925 (95%CI, 0.855-0.994) in the external validation cohort.

The radiomics signature consisting of 10 radiomics features in our study was able to differentiate SDM ccRCC from without SDM ccRCC with acceptable performance in the internal validation (0.869 [95%CI, 0.806-0.933]) and external validation (0.882[95%CI, 0.796-0.967]) cohorts. In our radiomics signature, most of the features were transformed by wavelet filter, which splits imaging data into different frequency components on three axis of the tumor region (19), indicating that the wavelet features may further explore the spatial heterogeneity at multiple scales within tumor regions. Some previous studies have reported that wavelet features might better reveal tumor biology and heterogeneity (20, 21). Liang et al. found that wavelet features were of great importance to predict early recurrence of intrahepatic cholangiocarcinoma after partial hepatectomy (20). The shape feature, Maximum 3D Diameter, defined as the largest pairwise Euclidean distance between tumor surface mesh vertices, had the highest weights in the radiomics model. The Maximum 3D Diameter was positively correlated with SDM ccRCC, suggesting that larger tumor may be seen more commonly in SDM ccRCC, which is consistent with previous studies (22, 23). Small Dependence Low Gray Level Emphasis (SDLGLE) is defined as the joint distribution of small dependence with lower gray-level values, and the greater value indicates less homogeneous textures and a greater concentration of low gray-level values in the image (24). We assumed the greater value of SDLGLE in SDM ccRCC might be related to the combination of a larger range of necrosis components with lower gray values (25, 26).

Our study took plenty of clinical factors into account. In line with previous studies, AFR was selected as an independent predictor for without SDM ccRCC, which suggested that ccRCC patients with decreased AFR are more likely to have SDM (27–29). Numerous experimental researches have convincingly supported the concept that inflammation is an imperative ingredient of tumor progression (30–32). Serum albumin has protective effects such as nutrition and anti-inflammatory, and fibrinogen can promote the invasion and metastasis of tumor cells through epithelial-mesenchymal transition and induce tumor blood vessel formation, thereby participating in tumor progression (33, 34). Therefore, decreased serum albumin and elevated fibrinogen are symptoms of elevated systemic inflammation, and decreased AFR might be connected with a worse prognosis (27). According to the equation for the Cli-score developed in our study, ccRCC with decreased AFR tended to be accompanied with SDM, which was consistent with the previous studies. It should be noted that, for the other clinical features associated with inflammation, including PLR, NLR and RLR, we found they were significantly different between SDM ccRCC and without SDM ccRCC in training cohort. However, these clinical features were not independent factors after multivariate analysis and were excluded in the final model. We presume that the difference in endpoint event and the unbalance of the two groups might explain the discrepancy between study results.

There are several limitations to our study. First, the retrospective nature might have inevitably introduced bias in population selection. The two groups in our study population was unbalanced, which might indicate a spectrum bias and might have influenced the diagnostic performance. Besides, there was an imbalance between the training and internal validation cohort, due to the relatively small sample size. Prospective multicenter studies with considerably large datasets are needed to further validate the robustness and reproducibility of our model. Second, owing to the limitation of the small number of SDM ccRCC, there is not enough data to differentiate various site of SDM to perform a stratified analysis, which is meaningful. A large-scale prospective study is needed to predict the exact location of SDM. Third, CT acquisition parameters and reconstruction techniques were not consistent due to the retrospective and multi-institutional nature of the study. Fourth, not all SDM were diagnosed with pathologic examination. Some lesions were diagnosed with typical radiologic findings and follow-up imaging. Finally, different observers for segmentation could have affected the stability of radiomics features. Although only features with ICCs greater than 0.75 were kept for radiomics signature construction in our study, automated and accurate tumor segmentation must be developed to facilitate the efficiency of the radiomics process. In addition, it would be more interesting to develop a model to predict metachronous disease, which could be helpful in managing follow-up schedule.

In conclusion, our study presented a CT-based radiomics nomogram that showed satisfactory performance in predicting SDM among ccRCC patients, which can enable physicians to make more informed diagnostic examinations and treatment decisions.

## Data availability statement

The raw data supporting the conclusions of this article will be made available by the authors, without undue reservation.

## Ethics statement

Written informed consent was not obtained from the individual(s) for the publication of any potentially identifiable images or data included in this article.

## Author contributions

Study conception and design: XY, LG, SZ, BK, and XW. Administrative support: XW. Provision of study materials or patients: XY, LG, CS, and BK. Collection and assembly of data: XY, LG, and SZ. Data analysis and interpretation: XY, LG, JZ, and BK. All authors contributed to the article and approved the submitted version.
