# Corrigendum: Development and validation of A CT-based radiomics nomogram for prediction of synchronous distant metastasis in clear cell renal cell carcinoma

**DOI:** 10.3389/fonc.2023.1193426

**Published:** 2023-04-12

**Authors:** Xinxin Yu, Lin Gao, Shuai Zhang, Cong Sun, Juntao Zhang, Bing Kang, Ximing Wang

**Affiliations:** ^1^ School of Medicine, Shandong University, Jinan, China; ^2^ Department of Radiology, Shandong Provincial Hospital Affiliated to Shandong First Medical University, Jinan, China; ^3^ Department of Nuclear Medicine, The First Affiliated Hospital of Shandong First Medical University & Shandong Provincial Qianfoshan Hospital, Jinan, China; ^4^ School of Medicine, Shandong First Medical University, Jinan, China; ^5^ GE Healthcare, PDx GMS Advanced Analytics, Shanghai, China

**Keywords:** radiomics, nomogram, clear cell renal cell carcinoma, metastasis, computed tomography

In the published article, there was an error in the order of the affiliations 1 and 2. Instead of “1Department of Radiology, Shandong Provincial Hospital Affiliated to Shandong First Medical University, Jinan, China; 2School of Medicine, Shandong University, Jinan, China”, it should be “1School of Medicine, Shandong University, Jinan, China; 2Department of Radiology, Shandong Provincial Hospital Affiliated to Shandong First Medical University, Jinan, China”. Additionally, the correct affiliation for authors Cong Sun and Bing Kang is 2 instead of 1.

The authors apologize for this error and state that this does not change the scientific conclusions of the article in any way. The original article has been updated.

