# Supplementary material for: Development and validation of A CT-based radiomics nomogram for prediction of synchronous distant metastasis in clear cell renal cell carcinoma
Source: Front Oncol. 2023 Jan 4;12:1016583. doi: 10.3389/fonc.2022.1016583 (PMC9846314; doi:10.3389/fonc.2022.1016583)
Supplement: Supplementary file 1 [file DataSheet_1.doc]

**Supplementary Methods**

**Supplementary S1: The inclusion and exclusion criteria**

The inclusion criteria were as follows: (a) preoperative contrast-enhanced CT within two weeks before surgery; (b) SDM was confirmed by pathology, or follow-up; (c) the patients without SDM were confirmed by no signs of distant metastases through whole-body imaging examinations before nephrectomy or no suspicious metastasis during a period of follow up for at least 6 months after surgery.

The exclusion criteria included: (a) patients with multiple renal tumors; (b) patients accompanied by other malignant tumors besides ccRCC; (c) patients with history of previous anticancer therapy for renal lesion; (d) patients without any follow-up information.

**Supplementary S2: CT Image Acquisition Parameters**

The cases in Shandong Provincial Hospital Affiliated to Shandong First Medical University underwent contrast-enhanced CT using the multidetector row CT systems (Aquilion ONE, TOSHIBA; Discovery 750, GE Healthcare; Somatom Definition Flash, Siemens Healthcare). The acquisition parameters are as follows: 120 kV tube voltage, 250-400 mA (using automatic tube current modulation technique) tube current, 0.5 s or 0.6 s gantry rotation time, 80×0.5 mm or 64 × 0.625 mm detector collimation, a matrix of 512×512, a pitch of 1.388 or 0.984, and a soft reconstruction kernel. Axial images were reconstructed with a 5 mm slice thickness. An 80-90 mL volume of iodinated contrast medium (Omnipaque 350, GE Healthcare, Shanghai, China) was injected into the antecubital vein by a power injector (Ulrich CT Plus 150, Ulrich Medical, Ulm, Germany) at a rate of 3.0 mL/s. Following unenhanced CT images, the corticomedullary phase (CMP, 30s) and nephrographic phase (NP, 90s) were obtained for all patients.

The patients in Shandong Medical Imaging Research Institute were examined using 64–detector row CT scanner (Somatom Definition Flash, Siemens Healthcare) using the following parameters: 0.5 s gantry rotation time, 120 kV tube voltage, 250-400 mA (using automatic tube current modulation technique) tube current, 128×0.6 mm detector collimation, a matrix of 512×512, a pitch of 0.8, and a soft reconstruction kernel. Axial images were reconstructed with a 5 mm slice thickness. An 80 mL volume of iodinated contrast medium (Iohexol Injection 350, Starry Pharmaceutical Co., Shanghai, China) was injected into the antecubital vein by a power injector (Bayer Drive, Indianola, U.S.A.) at a rate of 3.0 mL/s. Following unenhanced CT images, the corticomedullary phase (CMP, 30s) and nephrographic phase (NP, 90s) were obtained.

**Supplementary S3: Radiomic Feature Extraction Methodology**

A total of 1409 quantitative radiomics features were extracted from CT images with Radcloud platform, these features can be grouped into three groups. (1) First-order features (n = 18), described the intensity information in the region of interest, such as mean, standard deviation, variance, maximum, median, range, etc. (2) Size- and shape-based features (n = 14), which reflected the size and shape of the region, such as volume, surface area, compactness, maximum diameter, etc. (3) Texture features, which could quantify regional heterogeneity differences, such as gray-level co-occurrence matrix (GLCM, n = 24), gray-level size zone matrix (GLSZM, n = 16), gray-level dependence matrix (GLDM, n = 14), neighbouring gray tone difference matrix, (NGTDM, n=5), and gray-level run-length matrix (GLRLM, n = 16). Another 1302 features, including the first-order statistics and texture features derived from filter of the original images: logarithm, exponential, gradient, square, square root, local binary patterns (LBP), the wavelet transform decomposes the tumor area image into low-frequency components (L) or high-frequency components (H) in the three directions of the x, y, and z axes. Eight types of wavelet features were obtained and labeled as LLL, LLH, LHL, LHH, HLL, HLH, HHL, HHH according to their different decomposition orders.

**Supplementary Figure S1**


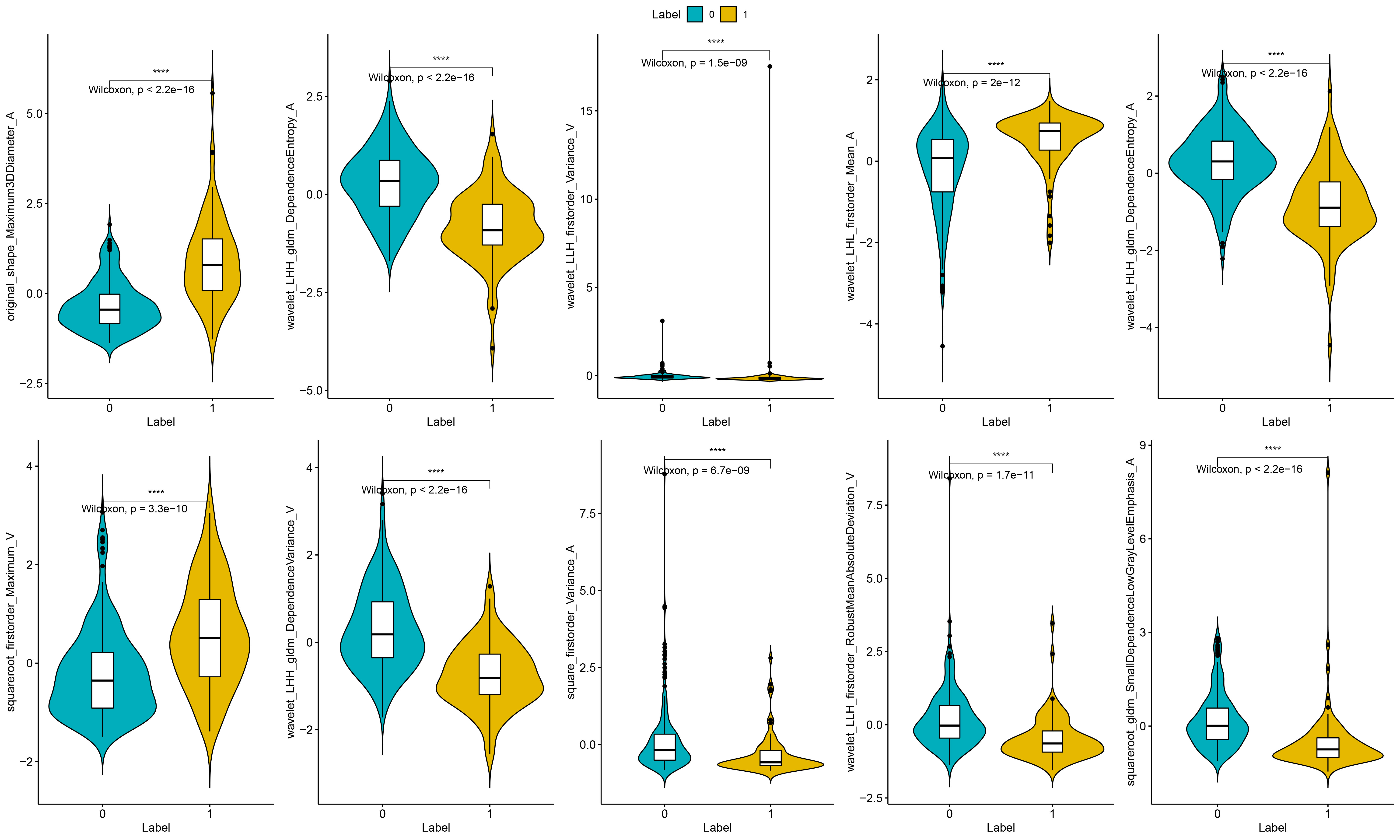


Figure S1 The difference of the 10 radiomics features between the SDM ccRCC and without SDM ccRCC groups
